# Propyl 2-(4-methyl­benzene­sulfonamido)­benzoate

**DOI:** 10.1107/S1600536812015528

**Published:** 2012-04-13

**Authors:** Ghulam Mustafa, Tahir Muhmood, Islam Ullah Khan, Mehmet Akkurt

**Affiliations:** a Department of Chemistry, GC University, Lahore 54000, Pakistan; b Department of Physics, Faculty of Sciences, Erciyes University, 38039 Kayseri, Turkey

## Abstract

In the title compound, C_17_H_19_NO_4_S, the terminal ethyl group is disordered over two sets of sites, with refined site occupancies of 0.536 (7) and 0.464 (7). The dihedral angle between the two aromatic rings is 81.92 (12)°. The mol­ecular conformation is stabilized by intra­molecular N—H⋯O and C—H⋯O hydrogen bonds, which generate *S*(6) motifs. In the crystal, mol­ecules are linked by C—H⋯O hydrogen bonds, forming chains along the *b* axis.

## Related literature

For related structures, see: Mustafa *et al.* (2010 ▶, 2011 ▶, 2012 ▶); Khan *et al.* (2011 ▶). For bond-length data, see: Allen *et al.* (1987 ▶).
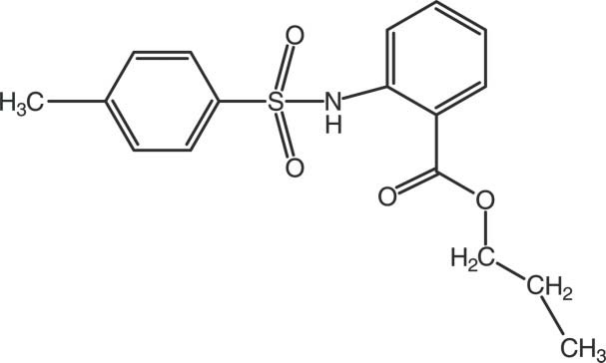


## Experimental

### 

#### Crystal data

C_17_H_19_NO_4_S
*M* *_r_* = 333.40
Monoclinic, 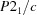

*a* = 16.206 (5) Å
*b* = 8.513 (2) Å
*c* = 12.021 (3) Å
β = 92.352 (2)°
*V* = 1657.0 (8) Å^3^
*Z* = 4
Mo *K*α radiation
μ = 0.22 mm^−1^
*T* = 296 K
0.34 × 0.22 × 0.21 mm

#### Data collection

Bruker APEXII CCD diffractometer
15110 measured reflections
4039 independent reflections
2343 reflections with *I* > 2σ(*I*)
*R*_int_ = 0.071

#### Refinement

*R*[*F* ^2^ > 2σ(*F* ^2^)] = 0.056
*wR*(*F* ^2^) = 0.178
*S* = 0.95
4039 reflections
209 parameters
4 restraints
H-atom parameters constrained
Δρ_max_ = 0.41 e Å^−3^
Δρ_min_ = −0.37 e Å^−3^

### 

Data collection: *APEX2* (Bruker, 2007 ▶); cell refinement: *SAINT* (Bruker, 2007 ▶); data reduction: *SAINT*; program(s) used to solve structure: *SIR97* (Altomare *et al.*, 1999 ▶); program(s) used to refine structure: *SHELXL97* (Sheldrick, 2008 ▶); molecular graphics: *ORTEP-3 for Windows* (Farrugia, 1997 ▶) and *PLATON* (Spek, 2009 ▶); software used to prepare material for publication: *WinGX* (Farrugia, 1999 ▶) and *PLATON*.

## Supplementary Material

Crystal structure: contains datablock(s) global, I. DOI: 10.1107/S1600536812015528/hg5208sup1.cif

Structure factors: contains datablock(s) I. DOI: 10.1107/S1600536812015528/hg5208Isup2.hkl

Supplementary material file. DOI: 10.1107/S1600536812015528/hg5208Isup3.cml

Additional supplementary materials:  crystallographic information; 3D view; checkCIF report

## Figures and Tables

**Table 1:** Hydrogen-bond geometry (Å, °)

*D*—H⋯*A*	*D*—H	H⋯*A*	*D*⋯*A*	*D*—H⋯*A*
N1—H1⋯O3	0.86	2.11	2.643 (3)	119
C10—H10⋯O1^i^	0.93	2.49	3.391 (3)	163
C9—H12⋯O2	0.93	2.36	3.027 (3)	128

Symmetry code: (i) 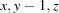.

**Fig. 1.**
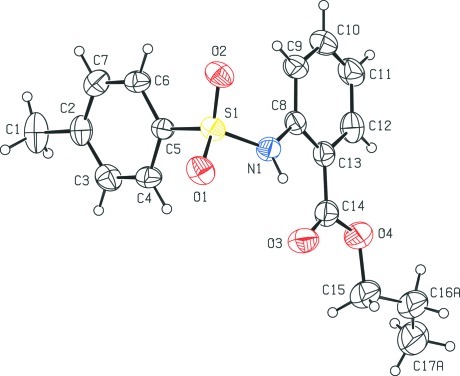
The molecular structure of (I) with the atom numbering scheme. Displacement ellipsoids for non-H atoms are drawn at the 30% probability level. Only the atoms of major disorder component are shown.

**Fig. 2.**
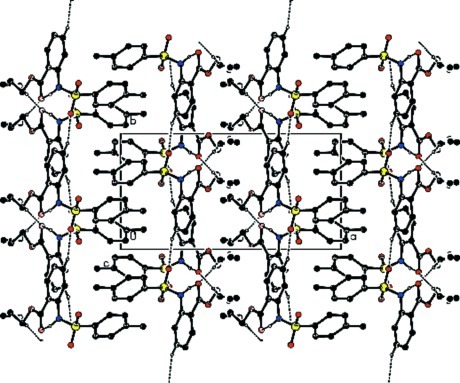
View of the hydrogen-bonding interactions of (I) along the c axis in the unit cell. Only the hydrogen atoms involved in hydrogen bonds (dotted lines) are drawn, for clarity.

## supplementary crystallographic information

### Comment

As part of our ongoing studies of sulfonamides with potential biological properties (Mustafa *et al.*, 2010, 2011, 2012; Khan *et al.*, 2011), we now describe the title compound, propyl 2-{[(4-methylphenyl)sulfonyl]amino}benzoate, (I).

In the title molecule (I), (Fig. 1), the dihedral angle between the two benzene rings (C2—C7) and (C8—C13) is 81.92 (12)°. The C—S—N—C torsion angle is 61.6 (2)°. All the bond lengths (Allen *et al.*, 1987) and angles are within normal ranges and are comparable to those found for similar structures (Mustafa *et al.*, 2010; 2011, 2012; Khan *et al.*, 2011).

The molecular conformation of (I) is stabilized by intramolecular N—H···O and C—H···O hydrogen-bond interactions, generating S(6) motifs (Table 1). The crystal structure is stabilized by C—H···O hydrogen bonds, forming chains along the *b* axis (Table 1, Fig. 2).

### Experimental

To an aqueous solution of *o*-amino benzoic acid (1.0 g, 7.3 mmol), sodium carbonate (1 N) was added to adjust the pH 8. Then *p*-toluenesulfonyl chloride (1.80 g, 9.48 mmol) was added and the mixture stirred at room temperature keeping the pH of the mixture up to 8.0 with occasional addition of sodium carbonate solution. Progress and completion of the reaction was confirmed by TLC and conversion of suspension into clear solution. After 2 h, whole mixture was poured into a beaker and the pH was adjusted to 2.0 by 1 N HCl. Precipitates were produced which were filtered and washed with distilled water.

The prepared sulfonamide (2-(Toluene-4-sulfonylamino)-benzoic acid) (1.0 g, 3.43 mmol), DMF (10 ml) and n-hexane washed sodium hydride (0.25 g, 10.31 mmol) were stirred at room temperature for 40 min followed by the addition of propyl iodide (0.76 g, 4.56 mmol). The whole reaction mixture was stirred till the completion of the reaction and poured into crushed ice in a beaker. The pH of the mixture was adjusted to 4.0 with 1 N HCl. Precipitates were produced, filtered, washed twice with distilled water and crystallized in chloroform.

### Refinement

All H atoms were positioned with idealized geometry and were refined using a riding model with N—H = 0.86 Å, C—H = 0.93, 0.96 or 0.97 Å, and with *U*_iso_(H) = 1.2 or 1.5*U*_eq_(C,*N*). The atoms of the terminal ethane group are disordered over two sets of sites, with refined site-occupancies of 0.536 (7): 0.464 (7). Five poorly fitted reflections (1 0 0), (-3 2 2), (8 0 2),(-3 6 1) and (-1 1 7) were omitted from the refinement.

### Crystal data

**Table:**

C_17_H_19_NO_4_S	*F*(000) = 704
*M**_r_* = 333.40	*D*_x_ = 1.337 Mg m^−^^3^
Monoclinic, *P*2_1_/*c*	Mo *K*α radiation, λ = 0.71073 Å
Hall symbol: -P 2ybc	Cell parameters from 4265 reflections
*a* = 16.206 (5) Å	θ = 2.5–23.7°
*b* = 8.513 (2) Å	µ = 0.22 mm^−^^1^
*c* = 12.021 (3) Å	*T* = 296 K
β = 92.352 (2)°	Needle, dark brown
*V* = 1657.0 (8) Å^3^	0.34 × 0.22 × 0.21 mm
*Z* = 4	

### Data collection

**Table:**

Bruker APEXII CCD diffractometer	2343 reflections with *I* > 2σ(*I*)
Radiation source: sealed tube	*R*_int_ = 0.071
Graphite monochromator	θ_max_ = 28.3°, θ_min_ = 2.5°
φ and ω scans	*h* = −21→21
15110 measured reflections	*k* = −11→9
4039 independent reflections	*l* = −16→16

### Refinement

**Table:**

Refinement on *F*^2^	Primary atom site location: structure-invariant direct methods
Least-squares matrix: full	Secondary atom site location: difference Fourier map
*R*[*F*^2^ > 2σ(*F*^2^)] = 0.056	Hydrogen site location: inferred from neighbouring sites
*wR*(*F*^2^) = 0.178	H-atom parameters constrained
*S* = 0.95	*w* = 1/[σ^2^(*F*_o_^2^) + (0.1068*P*)^2^] where *P* = (*F*_o_^2^ + 2*F*_c_^2^)/3
4039 reflections	(Δ/σ)_max_ < 0.001
209 parameters	Δρ_max_ = 0.41 e Å^−^^3^
4 restraints	Δρ_min_ = −0.37 e Å^−^^3^

### Special details

**Table:**

Geometry. Bond distances, angles *etc*. have been calculated using the rounded fractional coordinates. All su's are estimated from the variances of the (full) variance-covariance matrix. The cell e.s.d.'s are taken into account in the estimation of distances, angles and torsion angles
Refinement. Refinement on *F*^2^ for ALL reflections except those flagged by the user for potential systematic errors. Weighted *R*-factors *wR* and all goodnesses of fit *S* are based on *F*^2^, conventional *R*-factors *R* are based on *F*, with *F* set to zero for negative *F*^2^. The observed criterion of *F*^2^ > σ(*F*^2^) is used only for calculating -*R*-factor-obs *etc*. and is not relevant to the choice of reflections for refinement. *R*-factors based on *F*^2^ are statistically about twice as large as those based on *F*, and *R*-factors based on ALL data will be even larger.

### Fractional atomic coordinates and isotropic or equivalent isotropic displacement parameters (Å^2^)

**Table:**

	*x*	*y*	*z*	*U*_iso_*/*U*_eq_	Occ. (<1)
S1	0.80026 (4)	0.16805 (7)	0.29221 (4)	0.0584 (2)	
O1	0.77925 (11)	0.32859 (19)	0.31033 (16)	0.0767 (7)	
O2	0.81365 (11)	0.06631 (19)	0.38522 (12)	0.0750 (7)	
O3	0.64329 (12)	0.1994 (2)	0.03046 (17)	0.0869 (8)	
O4	0.59833 (12)	0.0193 (2)	−0.09050 (17)	0.0942 (8)	
N1	0.72438 (11)	0.1015 (2)	0.21272 (16)	0.0630 (7)	
C1	1.10575 (19)	0.1688 (4)	0.0220 (3)	0.1018 (14)	
C2	1.02933 (15)	0.1638 (3)	0.0893 (2)	0.0667 (9)	
C3	0.96043 (17)	0.2539 (3)	0.0577 (2)	0.0722 (10)	
C4	0.89084 (15)	0.2530 (3)	0.11718 (18)	0.0622 (8)	
C5	0.88847 (13)	0.1625 (2)	0.21260 (16)	0.0501 (7)	
C6	0.95491 (14)	0.0702 (3)	0.24444 (18)	0.0606 (8)	
C7	1.02450 (15)	0.0731 (3)	0.1834 (2)	0.0718 (9)	
C8	0.71875 (13)	−0.0507 (2)	0.16566 (19)	0.0558 (7)	
C9	0.75079 (16)	−0.1809 (3)	0.2218 (2)	0.0696 (9)	
C10	0.74210 (19)	−0.3271 (3)	0.1755 (3)	0.0821 (11)	
C11	0.70270 (19)	−0.3495 (3)	0.0752 (3)	0.0795 (11)	
C12	0.66964 (16)	−0.2228 (3)	0.0188 (2)	0.0763 (10)	
C13	0.67714 (13)	−0.0703 (3)	0.0631 (2)	0.0594 (8)	
C14	0.63951 (15)	0.0624 (4)	0.0015 (2)	0.0691 (10)	
C15	0.5563 (2)	0.1421 (5)	−0.1561 (3)	0.1144 (14)	
C16A	0.5014 (6)	0.0493 (10)	−0.2409 (6)	0.1144 (14)	0.536 (7)
C17A	0.4972 (7)	0.070 (2)	−0.3449 (9)	0.131 (4)	0.536 (7)
C16B	0.5434 (6)	0.0897 (12)	−0.2659 (7)	0.1144 (14)	0.464 (7)
C17B	0.4682 (7)	0.077 (3)	−0.3096 (12)	0.131 (4)	0.464 (7)
H1	0.68400	0.16460	0.19800	0.0760*	
H7	1.06970	0.01200	0.20630	0.0860*	
H5	0.84520	0.31260	0.09390	0.0750*	
H6	0.96210	0.31630	−0.00570	0.0870*	
H11	0.69810	−0.44980	0.04490	0.0950*	
H12	0.77820	−0.16880	0.29080	0.0830*	
H13	0.64190	−0.23790	−0.04960	0.0920*	
H14A	1.09050	0.15610	−0.05550	0.1530*	
H14B	1.14240	0.08550	0.04550	0.1530*	
H14C	1.13300	0.26800	0.03320	0.1530*	
H15A	0.59570	0.20750	−0.19340	0.1370*	
H15B	0.52310	0.20800	−0.10950	0.1370*	
H16A	0.51600	−0.06020	−0.22990	0.1370*	0.536 (7)
H16B	0.44540	0.06040	−0.21660	0.1370*	0.536 (7)
H17A	0.44610	0.02730	−0.37500	0.1970*	0.536 (7)
H17B	0.54260	0.01780	−0.37790	0.1970*	0.536 (7)
H17C	0.49940	0.18030	−0.36090	0.1970*	0.536 (7)
H8	0.95270	0.00630	0.30700	0.0730*	
H10	0.76380	−0.41340	0.21400	0.0980*	
H16C	0.57360	0.15980	−0.31310	0.1370*	0.464 (7)
H16D	0.56910	−0.01290	−0.27080	0.1370*	0.464 (7)
H17D	0.46750	0.00200	−0.36910	0.1970*	0.464 (7)
H17E	0.45030	0.17760	−0.33810	0.1970*	0.464 (7)
H17F	0.43160	0.04330	−0.25350	0.1970*	0.464 (7)

### Atomic displacement parameters (Å^2^)

**Table:**

	*U*^11^	*U*^22^	*U*^33^	*U*^12^	*U*^13^	*U*^23^
S1	0.0714 (4)	0.0507 (4)	0.0533 (3)	−0.0016 (3)	0.0056 (3)	−0.0040 (2)
O1	0.0920 (13)	0.0510 (11)	0.0879 (12)	0.0020 (8)	0.0141 (10)	−0.0185 (8)
O2	0.1016 (13)	0.0742 (12)	0.0496 (9)	−0.0102 (9)	0.0074 (9)	0.0070 (8)
O3	0.0933 (14)	0.0751 (14)	0.0913 (13)	0.0160 (10)	−0.0083 (11)	0.0078 (11)
O4	0.0951 (14)	0.1008 (16)	0.0846 (12)	−0.0095 (11)	−0.0226 (11)	0.0158 (11)
N1	0.0598 (11)	0.0503 (12)	0.0785 (13)	0.0065 (9)	−0.0010 (9)	−0.0059 (10)
C1	0.0773 (19)	0.131 (3)	0.099 (2)	−0.0341 (17)	0.0251 (17)	−0.043 (2)
C2	0.0646 (15)	0.0729 (17)	0.0628 (14)	−0.0159 (12)	0.0046 (11)	−0.0227 (13)
C3	0.0886 (19)	0.0786 (18)	0.0495 (12)	−0.0142 (14)	0.0033 (12)	0.0045 (12)
C4	0.0723 (15)	0.0624 (15)	0.0515 (12)	0.0043 (11)	−0.0038 (11)	0.0084 (10)
C5	0.0618 (12)	0.0434 (12)	0.0444 (10)	−0.0025 (9)	−0.0060 (9)	−0.0020 (8)
C6	0.0729 (15)	0.0562 (14)	0.0519 (12)	0.0057 (11)	−0.0061 (11)	0.0017 (10)
C7	0.0651 (15)	0.0727 (18)	0.0768 (16)	0.0088 (12)	−0.0077 (13)	−0.0115 (13)
C8	0.0532 (12)	0.0477 (13)	0.0668 (13)	−0.0045 (10)	0.0077 (10)	−0.0013 (10)
C9	0.0758 (16)	0.0529 (15)	0.0792 (16)	−0.0033 (12)	−0.0070 (13)	0.0034 (12)
C10	0.0897 (19)	0.0503 (16)	0.106 (2)	0.0007 (13)	0.0017 (17)	0.0060 (14)
C11	0.0877 (19)	0.0483 (16)	0.103 (2)	−0.0054 (13)	0.0099 (16)	−0.0103 (14)
C12	0.0714 (17)	0.0787 (19)	0.0791 (17)	−0.0157 (14)	0.0054 (13)	−0.0180 (15)
C13	0.0514 (12)	0.0585 (15)	0.0687 (14)	−0.0042 (10)	0.0088 (10)	−0.0021 (11)
C14	0.0604 (14)	0.0755 (19)	0.0714 (16)	−0.0047 (13)	0.0026 (12)	0.0028 (13)
C15	0.092 (2)	0.142 (3)	0.107 (2)	−0.0034 (17)	−0.0213 (17)	0.043 (2)
C16A	0.092 (2)	0.142 (3)	0.107 (2)	−0.0034 (17)	−0.0213 (17)	0.043 (2)
C17A	0.090 (7)	0.188 (6)	0.116 (7)	−0.010 (7)	0.012 (4)	0.050 (7)
C16B	0.092 (2)	0.142 (3)	0.107 (2)	−0.0034 (17)	−0.0213 (17)	0.043 (2)
C17B	0.090 (7)	0.188 (6)	0.116 (7)	−0.010 (7)	0.012 (4)	0.050 (7)

### Geometric parameters (Å, º)

**Table:**

S1—O1	1.4273 (18)	C16A—C17A	1.262 (13)
S1—O2	1.4239 (17)	C16B—C17B	1.312 (15)
S1—N1	1.628 (2)	C1—H14A	0.9600
S1—C5	1.753 (2)	C1—H14B	0.9600
O3—C14	1.218 (4)	C1—H14C	0.9600
O4—C14	1.320 (3)	C3—H6	0.9300
O4—C15	1.461 (4)	C4—H5	0.9300
N1—C8	1.415 (3)	C6—H8	0.9300
N1—H1	0.8600	C7—H7	0.9300
C1—C2	1.507 (4)	C9—H12	0.9300
C2—C3	1.395 (4)	C10—H10	0.9300
C2—C7	1.375 (3)	C11—H11	0.9300
C3—C4	1.360 (4)	C12—H13	0.9300
C4—C5	1.384 (3)	C15—H15A	0.9700
C5—C6	1.375 (3)	C15—H15B	0.9700
C6—C7	1.371 (3)	C16A—H16A	0.9700
C8—C9	1.387 (3)	C16A—H16B	0.9700
C8—C13	1.391 (3)	C16B—H16C	0.9700
C9—C10	1.368 (4)	C16B—H16D	0.9700
C10—C11	1.355 (5)	C17A—H17C	0.9600
C11—C12	1.371 (4)	C17A—H17A	0.9600
C12—C13	1.407 (4)	C17A—H17B	0.9600
C13—C14	1.470 (4)	C17B—H17D	0.9600
C15—C16A	1.543 (9)	C17B—H17E	0.9600
C15—C16B	1.401 (9)	C17B—H17F	0.9600
			
O1—S1—O2	119.49 (11)	C4—C3—H6	119.00
O1—S1—N1	104.10 (10)	C3—C4—H5	120.00
O1—S1—C5	108.30 (10)	C5—C4—H5	120.00
O2—S1—N1	109.66 (10)	C5—C6—H8	120.00
O2—S1—C5	108.11 (10)	C7—C6—H8	120.00
N1—S1—C5	106.47 (10)	C2—C7—H7	119.00
C14—O4—C15	117.5 (2)	C6—C7—H7	119.00
S1—N1—C8	126.02 (15)	C8—C9—H12	120.00
C8—N1—H1	117.00	C10—C9—H12	120.00
S1—N1—H1	117.00	C9—C10—H10	119.00
C1—C2—C7	122.2 (2)	C11—C10—H10	119.00
C1—C2—C3	120.3 (2)	C10—C11—H11	120.00
C3—C2—C7	117.5 (2)	C12—C11—H11	120.00
C2—C3—C4	121.7 (2)	C11—C12—H13	120.00
C3—C4—C5	119.4 (2)	C13—C12—H13	120.00
C4—C5—C6	120.1 (2)	O4—C15—H15A	111.00
S1—C5—C4	119.24 (16)	O4—C15—H15B	111.00
S1—C5—C6	120.69 (15)	C16A—C15—H15A	111.00
C5—C6—C7	119.5 (2)	C16A—C15—H15B	111.00
C2—C7—C6	121.8 (2)	H15A—C15—H15B	109.00
N1—C8—C13	119.02 (19)	C16B—C15—H15A	80.00
N1—C8—C9	121.4 (2)	C16B—C15—H15B	132.00
C9—C8—C13	119.6 (2)	C15—C16A—H16A	106.00
C8—C9—C10	119.9 (2)	C15—C16A—H16B	106.00
C9—C10—C11	121.8 (3)	C17A—C16A—H16A	106.00
C10—C11—C12	119.3 (2)	C17A—C16A—H16B	106.00
C11—C12—C13	120.9 (2)	H16A—C16A—H16B	106.00
C12—C13—C14	119.4 (2)	C15—C16B—H16D	107.00
C8—C13—C12	118.5 (2)	C17B—C16B—H16C	107.00
C8—C13—C14	122.0 (2)	C17B—C16B—H16D	107.00
O4—C14—C13	113.2 (3)	H16C—C16B—H16D	107.00
O3—C14—O4	121.6 (3)	C15—C16B—H16C	107.00
O3—C14—C13	125.3 (2)	H17A—C17A—H17C	109.00
O4—C15—C16A	103.5 (4)	H17B—C17A—H17C	110.00
O4—C15—C16B	109.2 (5)	C16A—C17A—H17A	109.00
C15—C16A—C17A	126.1 (9)	C16A—C17A—H17B	110.00
C15—C16B—C17B	120.3 (10)	C16A—C17A—H17C	110.00
C2—C1—H14A	109.00	H17A—C17A—H17B	109.00
C2—C1—H14B	109.00	C16B—C17B—H17D	110.00
C2—C1—H14C	109.00	C16B—C17B—H17E	109.00
H14A—C1—H14B	110.00	C16B—C17B—H17F	110.00
H14A—C1—H14C	109.00	H17D—C17B—H17E	109.00
H14B—C1—H14C	109.00	H17D—C17B—H17F	110.00
C2—C3—H6	119.00	H17E—C17B—H17F	109.00
			
O1—S1—N1—C8	175.93 (18)	C3—C4—C5—S1	177.35 (18)
O2—S1—N1—C8	−55.1 (2)	C4—C5—C6—C7	2.5 (3)
C5—S1—N1—C8	61.6 (2)	S1—C5—C6—C7	−177.21 (18)
O1—S1—C5—C4	−48.9 (2)	C5—C6—C7—C2	−1.3 (4)
O2—S1—C5—C4	−179.66 (17)	N1—C8—C13—C14	0.6 (3)
N1—S1—C5—C4	62.58 (19)	C9—C8—C13—C12	−0.7 (3)
O1—S1—C5—C6	130.81 (18)	C9—C8—C13—C14	177.9 (2)
O2—S1—C5—C6	0.0 (2)	C13—C8—C9—C10	0.7 (4)
N1—S1—C5—C6	−117.76 (18)	N1—C8—C13—C12	−178.0 (2)
C15—O4—C14—O3	−0.8 (4)	N1—C8—C9—C10	178.0 (2)
C15—O4—C14—C13	178.1 (2)	C8—C9—C10—C11	0.0 (4)
C14—O4—C15—C16A	−168.8 (4)	C9—C10—C11—C12	−0.8 (5)
S1—N1—C8—C9	34.1 (3)	C10—C11—C12—C13	0.9 (4)
S1—N1—C8—C13	−148.64 (18)	C11—C12—C13—C14	−178.8 (2)
C3—C2—C7—C6	0.1 (4)	C11—C12—C13—C8	−0.1 (4)
C1—C2—C3—C4	−179.4 (3)	C8—C13—C14—O3	2.9 (4)
C1—C2—C7—C6	179.6 (3)	C8—C13—C14—O4	−175.9 (2)
C7—C2—C3—C4	0.1 (4)	C12—C13—C14—O3	−178.5 (2)
C2—C3—C4—C5	1.1 (4)	C12—C13—C14—O4	2.7 (3)
C3—C4—C5—C6	−2.3 (3)	O4—C15—C16A—C17A	−129.7 (11)

### Hydrogen-bond geometry (Å, º)

**Table:**

*D*—H···*A*	*D*—H	H···*A*	*D*···*A*	*D*—H···*A*
N1—H1···O3	0.86	2.11	2.643 (3)	119
C6—H8···O2	0.93	2.53	2.902 (3)	104
C10—H10···O1^i^	0.93	2.49	3.391 (3)	163
C9—H12···O2	0.93	2.36	3.027 (3)	128
C12—H13···O4	0.93	2.35	2.681 (3)	101

Symmetry code: (i) *x*, *y*−1, *z*.
